# New nomograms to predict overall and cancer‐specific survival of angiosarcoma

**DOI:** 10.1002/cam4.4425

**Published:** 2021-11-16

**Authors:** Yuan‐Yuan Liu, Bu‐Shu Xu, Qiu‐Zhong Pan, De‐Sheng Weng, Xing Zhang, Rui‐Qing Peng

**Affiliations:** ^1^ Melanoma and Sarcoma Medical Oncology Unit State Key Laboratory of Oncology in South China Collaborative Innovation Center for Cancer Medicine Sun Yat‐Sen University Cancer Center Guangzhou China; ^2^ State Key Laboratory of Oncology in South China Sun Yat‐Sen University Cancer Center Guangzhou China

**Keywords:** angiosarcoma, nomogram, prognosis, SEER

## Abstract

**Objective:**

This study was designed to establish and validate promising and reliable nomograms for predicting the survival of angiosarcoma (AS) patients.

**Methods:**

The Surveillance, Epidemiology, and End Results database was queried to collect the clinical information of 785 AS patients between 2004 and 2015. Data were split into a training cohort (*n* = 549) and a validation cohort (*n* = 236) without any preference. Univariate *Cox* and multivariate *Cox* regression analyses were performed to analyze the clinical parameters. Independent prognostic factors were then identified. Two nomograms were constructed to predict overall survival (OS) and cancer‐specific survival (CSS) at 3 and 5 years. Finally, the models were evaluated using concordance indices (C‐indices), calibration plots, and decision curve analysis (DCA).

**Results:**

Based on the inclusion and exclusion criteria, 785 individuals were included in this analysis. Univariate and multivariate *Cox* regression analyses revealed that age, tumor size, and stage were prognostic factors independently associated with the OS of AS. Tumor site, tumor size, and stage were associated with the CSS of AS. Based on the statistical results and clinical significance of variables, nomograms were built. The nomograms for OS and CSS had C‐indices of 0.666 and 0.654, respectively. The calibration curves showed good agreement between the predictive values and the actual values. DCA also indicated that the nomograms were clinically useful.

**Conclusion:**

We established nomograms with good predictive ability that could provide clinicians with better predictions about the clinical outcomes of AS patients.

## Graphical abstract

We constructed new and reliable nomograms with the clinical information obtained from the Surveillance, Epidemiology, and End Results database. Internal and external validation results suggested that our models had good prediction effects.

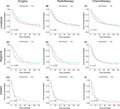

## 1 INTRODUCTION

Angiosarcoma (AS) is a rare but invasive type of soft tissue sarcoma (STS) that is derived from vascular and lymphatic endothelial cells^1^, ^2^ and accounts for approximately 1%–2% of STS.^3^, ^4^ It has a high rate of metastasis and recurrence.^5^ Current therapeutic strategies including surgery, chemotherapy, radiotherapy, and targeted therapy, have improved the prognosis of AS, but it still has a poor outcome.

Angiosarcoma is an uncommon tumor, and there are fewer relevant clinical research data and related literature on AS than on other tumors, such as liver cancer and lung cancer. Similarly, the prognostic models for patients with AS are scarce. Like other types of sarcomas, the main predicting outcome and staging methods of AS have based on the tumor‐node‐metastasis (TNM) staging system. The TNM staging system is of great value for clinical guidance, but there are still some inadequacies. For example, there may be differences in the handling of specimens between surgeons and pathologists, making staging more difficult. Moreover, the prognosis of patients with the same TNM stage and take the same treatment measures may vary greatly. Clinically, the accurate prediction of the prognosis in patients with AS is challenging, but it is also required. At present, there are no effective means to accurately predict the prognosis of patients with AS. Therefore, further studies on the construction of precision medicine tools for predicting the prognosis of AS are crucial.

Nomogram is a reliable and widely adopted method that is applied to assess the prognosis of tumors.^6^, ^7^ It enables personalized computation of outcomes based on clinical and pathological characteristics of both patient and tumor. The application of nomograms is convenient and economical, and most importantly, it has clinical applicability. Currently, nomogram has proven to be an effective method for predicting survival outcomes in sarcomas and other types of tumors.^8^, ^9^ However, as far as we know, there is still no report on the use of nomograms to predict the survival of AS that occurs in various parts of the body. Therefore, our research aims to develop reasonable and effective nomograms to help predict the prognosis of AS.

In this study, we analyzed the clinical information of AS patients from the Surveillance, Epidemiology, and End Results (SEER) database, a cancer statistics database in the United States. This study randomized 785 patients in a 7:3 ratio to training cohort and validation cohort. Based on the *Cox* regression analyses, we established new and reliable nomograms to assess the survival of patients with AS. Although AS is rare, we included sufficient samples to ensure the accuracy and validity of the models. In contrast with the SEER stage system, our nomograms were more intuitive, convenient, and individualized. Furthermore, our nomograms showed a better prediction of clinical outcomes than the SEER stage system for both overall survival (OS) and cancer‐specific survival (CSS).

## 2 METHODS

### 2.1 Source data and screening criteria

Data in this study were derived from the SEER database (https://seer.cancer.gov). This database is an authoritative data source of various clinical information about cancer survival and incidence in the US. Currently, it gathers and publishes survival and incidence data of various cancers from population‐based cancer registries, which cover almost 35% of the U.S. population.^10^ All study samples were collected from the public database and obtained by informed consent. Thus, no ethics review was required.

Inclusion criteria: (1) There was a definitive clinical and pathologic diagnosis. The histologic type and primary site were coded according to ICD‐O‐3/WHO 2008 (9120). (2) Patients were diagnosed with AS between 2004 and 2015. (3) Patients had complete survival information and follow‐up information. Exclusion criteria: (1) Patients' detailed clinical information, such as tumor size, and stage, was incomplete. (2) Treatments consisting of chemotherapy, surgery, and radiotherapy information were inadequate. (3) Those diagnosed with more than one primary tumor (Figure S1).

### 2.2 Clinical characteristics

For each case, we obtained the following clinical characteristics from the SEER database: race, sex, age at diagnosis, tumor site, stage, tumor size, surgery, radiotherapy, chemotherapy, survival time, and survival status. The cutoff point of tumor size was 50 mm and was divided into two groups: <50 mm and ≥50 mm. Likewise, age at diagnosis was grouped into two groups: <65 years and ≥65 years. The tumor site was split into two groups (trunk and limbs) according to the primary location of the tumors.^11^ In particular, primary head and neck tumors were more common, and they were included in the trunk group.^12^ Furthermore, stage was defined as “localized,” “regional,” or “distant.” In this retrospective study, the primary research endpoints were OS and CSS. OS was defined as the time from pathological diagnosis to the time of death as a result of any cause. CSS is the probability of surviving cancer in the absence of other causes of death. For cases lost to follow‐up before their death, the time of the last follow‐up was usually perceived as the date of death.

### 2.3 Nomograms' construction and validation

Eligible patients were randomly allocated in a 7:3 ratio to the training cohort or validation cohort. The training group contained 549 patients, and there were 236 patients in the verification group. In the training group, univariate *Cox* regression analysis was performed on each variable to identify the potential prognostic factors. Then, the independent prognostic factors of AS patients were assessed in multivariate *Cox* regression analysis based on a *p*‐value < 0.05. After that, nomograms were constructed for predicting the 3‐ and 5‐year OS and CSS using the rms package in R version 4.0.2. The training cohort and validation cohort underwent internal and external validation, respectively. The C‐indices were used to assess the accuracy and viability of our models. If the C‐index value was 0.5, it indicated that there was no difference, while 1.0 indicated perfect prediction. Calibration plots were also taken to evaluate the efficacy of the nomograms. This result indicated that the model was almost accurate when the predicted value was at 45° of the calibration plots. Finally, decision curve analysis (DCA) was used to compare the clinical prediction capabilities of the nomograms with the SEER stage.

### 2.4 Statistical analyses

The Kaplan–Meier method was used to calculate the survival curves, and the difference between the curves was evaluated by the log‐rank test. The chi‐square and Fisher exact tests were conducted for variables in the training cohort and validation cohort using SPSS version 25.0 (IBM Corporation). *Cox* regression analyses, nomogram development, and calibration curve calculation were performed using R version 4.0.2 (http://www.R‐project.org). All statistical tests were two‐sided. *p*‐values < 0.05 were considered statistically significant.

## 3 RESULTS

### 3.1 Clinical variables of the two cohorts

On the basis of the inclusion and exclusion criteria, 785 AS patients were selected from the SEER database, and samples were then randomly split into the training subset (70%) and the validation subset (30%). The patients' clinical variables in the two subsets were compared and are presented in Table 1. We performed a chi‐square test on the clinical information of the training group and the validation group, and all *p*‐values were >0.05. This result indicated that there was no significant difference between the two groups of clinical information.

**TABLE 1:** Clinicopathological characteristics of angiosarcoma

Characteristics	Training cohort (*n* = 549)	Validation cohort (*n* = 236)		Total (*n* = 785)	*p*‐value
Race					0.057
White	449 (81.8%)	194 (82.2%)		643 (81.9%)	
Black	47 (8.6%)	29 (12.3%)		76 (9.7%)	
Other^a^	53 (9.6%)	13 (5.5%)		66 (8.4%)	
Age (years)					0.528
<65	224 (40.8%)	102 (43.2%)		326 (41.5%)	
≥65	325 (59.2%)	134 (56.8%)		459 (58.5%)	
Gender					0.486
Male	255 (46.4%)	116 (49.2%)		371 (47.3%)	
Female	294 (53.6%)	120 (50.8%)		414 (52.7%)	
Tumor site					0.418
Trunk	412 (75.0%)	175 (74.2%)		587 (74.8%)	
Limbs	137 (25.0%)	61 (25.8%)		198 (25.2%)	
Tumor size (mm)					0.423
<50	273 (49.7%)	110 (46.6%)		383 (48.8%)	
≥50	276 (50.3%)	126 (53.4%)		402 (51.2%)	
Stage					0.784
Localized	305 (55.6%)	131 (55.5%)		436 (55.5%)	
Regional	140 (25.5%)	56 (23.7%)		196 (25.0%)	
Distant	104 (18.9%)	49 (20.8%)		153 (19.5%)	
Surgery					0.84
Yes	152 (27.7%)	67 (28.4%)		219 (27.9%)	
No/unknown	397 (72.3%)		169 (71.6%)	566 (72.1%)	
Radiotherapy					0.558
Yes	195 (35.5%)		89 (37.7%)	284 (36.2%)	
No/unknown	354 (64.5%)		147 (62.3%)	501 (63.8%)	
Chemotherapy					0.232
Yes	206 (37.5%)		78 (33.1%)	284 (36.2%)	
No/unknown	343 (62.5%)		158 (66.9%)	501 (63.8%)	

^a^ Other, American Indian/AK Native, Asian/Pacific Islander, other unspecified, unknown.

### 3.2 Survival analysis in the training and validation cohorts

**FIGURE 1:**
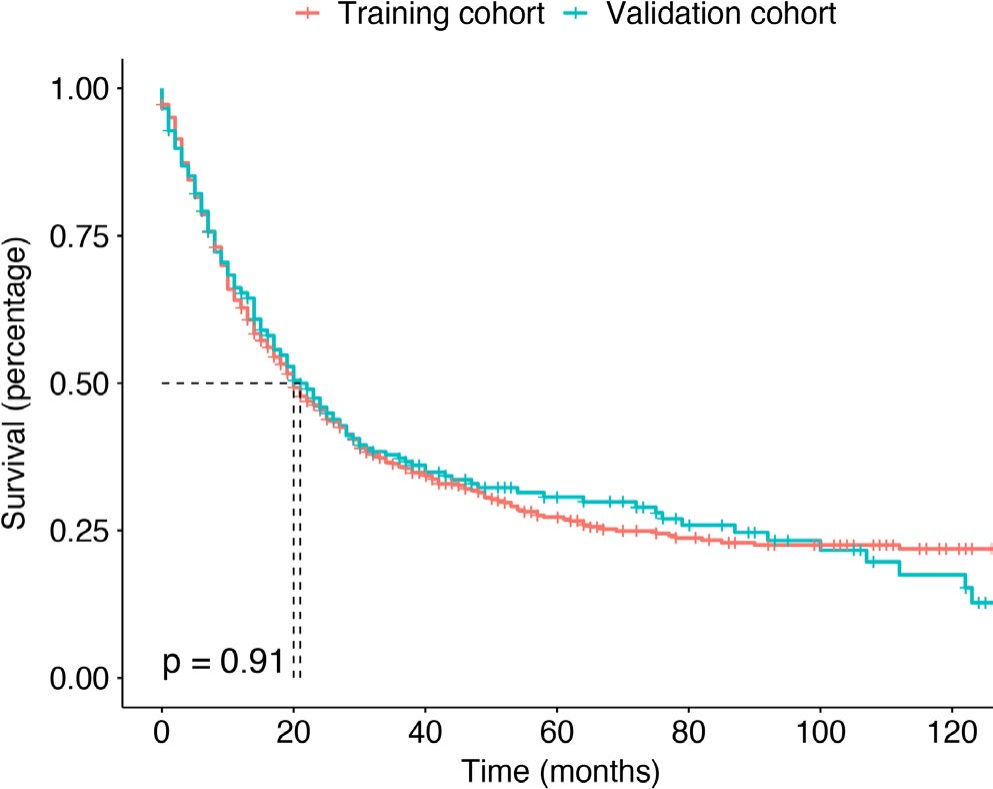
Survival analysis of AS patients in the training and validation cohorts. AS, angiosarcoma

In the training and validation cohorts, the median OS time of AS was 20 (95% confidence interval [CI]: 18–24) months and 21 (95% CI: 17–27) months, respectively. Differences in outcomes between the two subsets were not statistically significant. The results are shown in Figure 1.

### 3.3 Comparison of various treatment methods at different stages of AS

**FIGURE 2:**
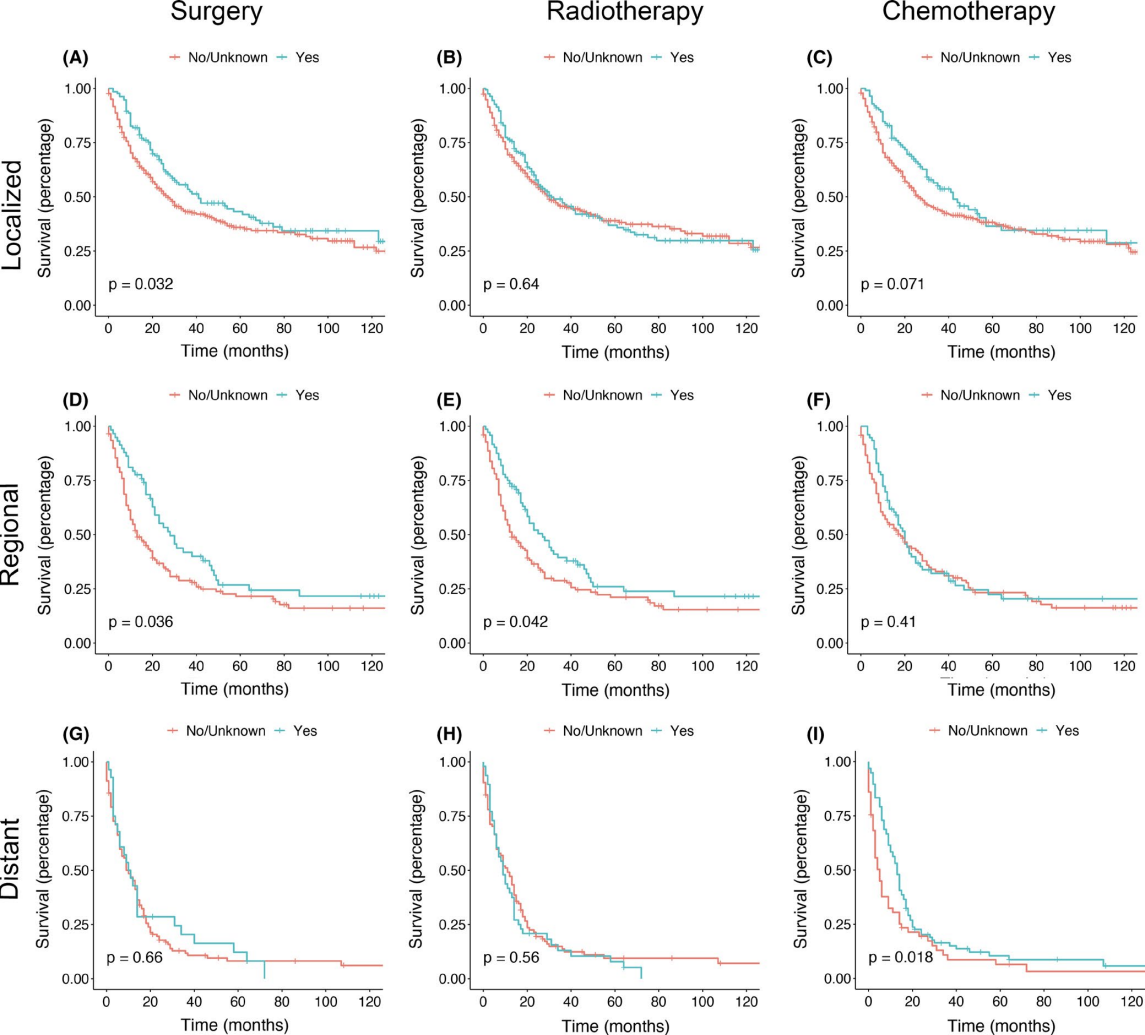
Kaplan–Meier diagrams of surgery (A, D, G), radiotherapy (B, E, H), and chemotherapy (C, F, I) in each clinical stage of angiosarcoma patients

As shown in Figure 2, surgery has made progress in the localized and regional stages. Chemotherapy had a good therapeutic effect in the distant stages, while radiotherapy showed good results only in the regional stage.

### 3.4 Univariate and multivariate analysis

Univariate *Cox* regression analysis was performed to evaluate the relationship between the clinical characteristics and prognosis of AS patients. As shown in Tables 2 and 3, the results indicated that age, tumor size, stage, and surgery were prognostic‐related risk factors (*p* < 0.05) for OS while race, sex, tumor site, radiation, and chemotherapy were not (*p* > 0.05). Age, tumor site, tumor size, and stage were the prognostic‐related risk factors (*p* < 0.05) for CSS. Furthermore, we performed multivariate *Cox* regression analysis with the clinical variables based on the risk factors above. The results suggested that age, stage, and tumor size were independent prognostic factors for OS in AS patients. Tumor site, tumor size, and stage were independent prognostic factors for CSS.

**TABLE 2:** Univariate and multivariate *Cox* regression analyses of OS in training cohort

Characteristic	Univariate analyses		Multivariate analyses	
	HR (95% CI)	*p*‐value	HR (95% CI)	*p*‐value
Race		0.648		
White	0.847 (0.599–1.196)			
Black	Reference			
Other^a^	0.844 (0.526–1.354)			
Age (years)		**<0.001** ^***^		
<65	**Reference**		**Reference**	
≥65	**1.527 (1.236–1.888)**		**1.788 (1.440–2.212)**	**<0.001** ^***^
Gender		0.223		
Male	1.134 (0.926–1.388)			
Female	Reference			
Tumor site		0.178		
Trunk	0.852 (0.677–1.073)			
Limbs	Reference			
Tumor size (mm)		**<0.001** ^***^		
<50	**Reference**		**Reference**	
≥50	**1.738 (1.415–2.134)**		**1.603 (1.295–1.986)**	**<0.001** ^***^
Stage		**<0.001** ^***^		
Localized	**0.407 (0.316–0.525)**		**0.435 (0.333–0.568)**	**<0.001** ^***^
Regional	**0.566 (0.426–0.750)**		**0.597 (0.447–0.796)**	**<0.001** ^***^
Distant	**Reference**		**Reference**	
Surgery		**0.011** ^*^		
Yes	**0.747 (0.594–0.941)**		0.823 (0.651–1.039)	0.102
No/unknown	**Reference**		Reference	
Radiotherapy		0.215		
Yes	0.875 (0.708–1.082)			
No/unknown	Reference			
Chemotherapy		0.697		
Yes	0.960 (0.779–1.182)			
No/unknown	Reference			

Statistically significant results were displayed with bold values. And the statistical significance was indicated as *p‐value < 0.05, **p‐value < 0.01, or ***p‐vulue < 0.001. Abbreviations: CI, confidence interval; HR, hazard ratio; OS, overall survival.

^a^ Other, American Indian/AK Native, Asian/Pacific Islander, other unspecified, unknown.

**TABLE 3:** Univariate and multivariate *Cox* regression analyses of CSS in training cohort

Characteristic	Univariate analyses		Multivariate analyses	
	HR (95% CI)	*p*‐value	HR (95% CI)	*p*‐value
Race		0.265		
White	0.835 (0.543–1.282)			
Black	Reference			
Other^a^	1.193 (0.668–2.131)			
Age (years)		**<0.001** ^***^		
<65	**Reference**		Reference	
≥65	**1.527 (1.236–1.888)**		0.957 (0.714–1.282)	0.768
Gender		0.107		
Male	1.248 (0.953–1.634)			
Female	Reference			
Tumor site		**0.004** ^**^		
Trunk	**0.634 (0.470–0.855)**		**0.519 (0.478–0.887)**	**0.006** ^**^
Limbs	**Reference**		**Reference**	
Tumor size (mm)		**<0.001** ^***^		
<50	**Reference**		**Reference**	
≥50	**1.738 (1.415–2.134)**		**1.654 (1.219–2.246)**	0.001^**^
Stage		**<0.001** ^***^		
Localized	**0.367 (0.265–0.508)**		**0.410 (0.288–0.585)**	**<0.001** ^***^
Regional	**0.480 (0.336–0.685)**		**0.519 (0.359–0.750)**	**<0.001** ^***^
Distant	**Reference**		**Reference**	
Surgery		0.071		
Yes	0.761 (0.562–1.031)			
No/unknown	Reference			
Radiotherapy		0.317		
Yes	0.868 (0.656–1.147)			
No/unknown	Reference			
Chemotherapy		0.222		
Yes	1.184 (0.903–1.553)			
No/unknown	Reference			

Statistically significant results were displayed with bold values. And the statistical significance was indicated as *p‐value < 0.05, **p‐value < 0.01, or ***p‐vulue < 0.001. Abbreviations: CI, confidence interval; CSS, cancer‐specific survival; HR, hazard ratio.

^a^ Other, American Indian/AK Native, Asian/Pacific Islander, other unspecified, unknown.

### 3.5 Construction and validation of the nomograms

**FIGURE 3:**
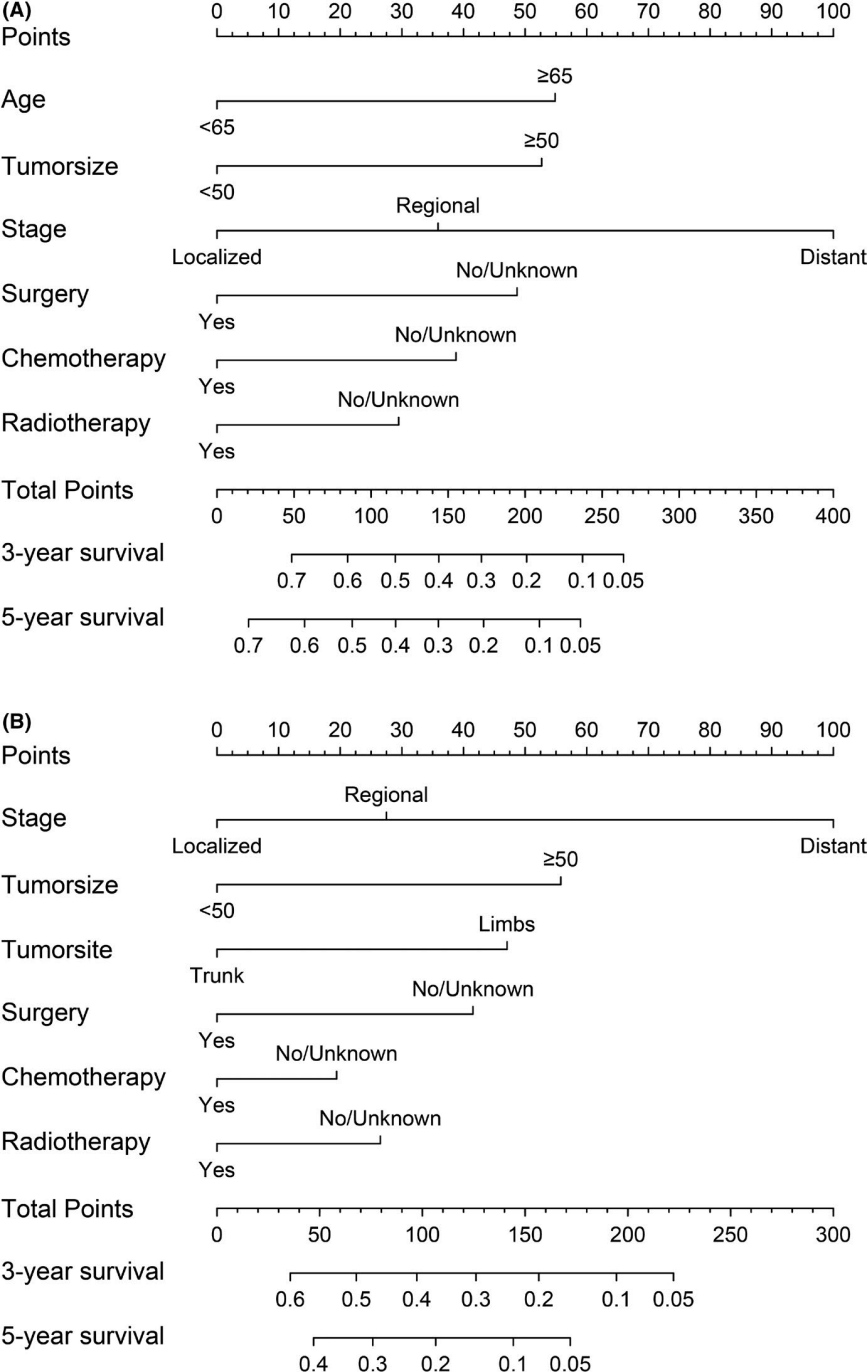
The chart signifies nomograms to predict the OS (A) and CSS (B) of AS patients. In the nomogram, a straight line is drawn perpendicular to the points line from the predictor state to obtain the corresponding score. The total points are from the addition of each indicator's points. Then, a straight line is drawn perpendicular to the line segment of Total Points downward from Total Points, and the value that intersects with Linear Predictor is the linear prediction value. Then the corresponding expected survival rate is obtained. Age measurement unit: year. Tumor size measurement unit: millimeter. AS, angiosarcoma; CSS, cancer‐specific survival; OS, overall survival

**FIGURE 4:**
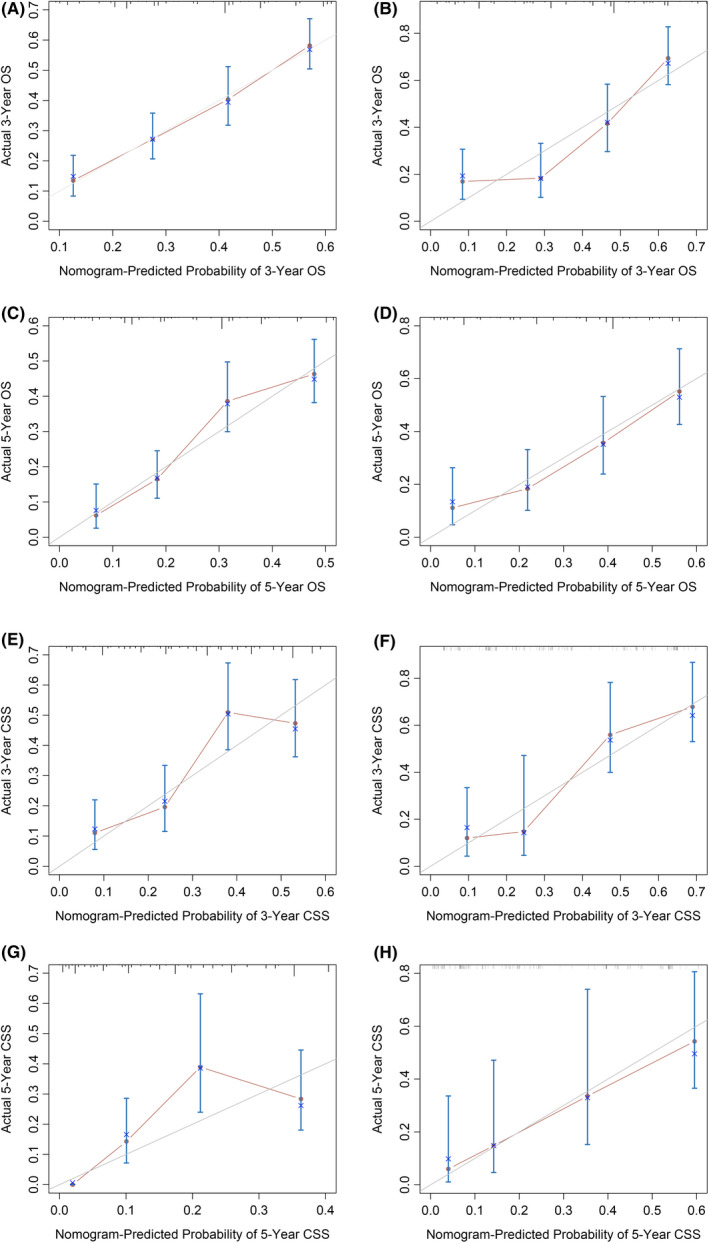
The first and second rows of the graphs show the internally and externally verified calibration charts of the actual 3‐ (A, B) and 5‐year (C, D) OS of the training and validation cohorts, respectively. Similarly, pictures in the last two rows reveal the internally and externally verified calibration charts of the actual 3‐ (E, F) and 5‐year (G, H) CSS of the training and validation cohorts. The predicted OS and CSS probabilities of the nomogram are shown on the X‐axis while the Y‐axis represents the actual survival. This indicates a higher prediction accuracy when the prediction falls on diagonal 45 in the calibration chart. CSS, cancer‐specific survival; OS, overall survival

**FIGURE 5:**
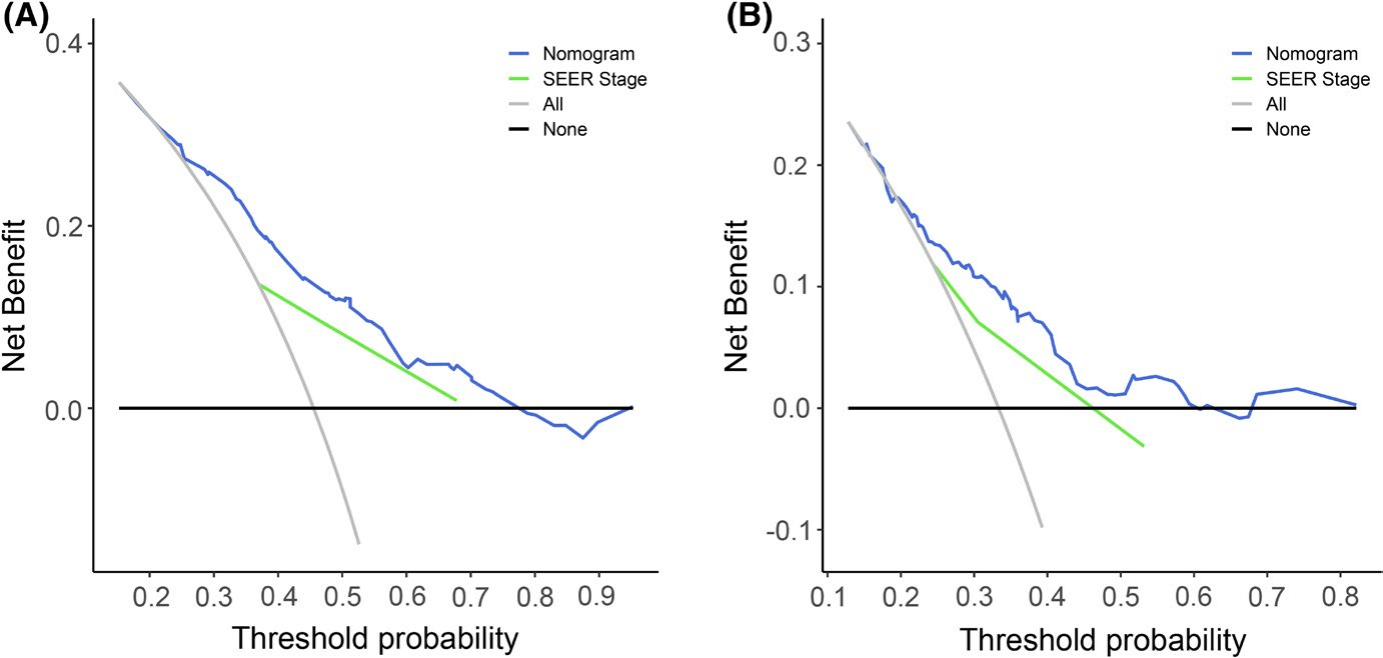
DCA of the nomograms for OS (A) and CSS (B). CSS, cancer‐specific survival; DCA, decision curve analysis; OS, overall survival; SEER, Surveillance, Epidemiology, and End Results

With the results in Tables 2 and 3, and combining the results of single factor *Cox* regression analysis, the limitations of SEER database and actual clinical significance of variables, we incorporated surgery, chemotherapy, and radiotherapy into the nomograms (Figure 3). A total score could be obtained by adding the scores obtained for each predicted value in the graph. For each patient, we calculated the survival probability with the total score. The C‐index for OS of AS are 0.666 (95% CI: 0.638–0.694). The C‐index for CSS of AS are 0.654 (95% CI: 0.613–0.696) (Table S1). Internal verification was performed through the training cohort while external verification was performed through the validation cohort. In addition, internal and external calibration curves reflected the excellent performance of the nomograms in predicting the 3‐, 5‐year OS in both training and validation cohorts, and 5‐year CSS in validation cohort (Figure 4). Specifically, the DCA results also indicated that if patient's threshold probability was >25%, the developed nomograms and the SEER stage were comparable in predicting the net benefits of OS and CSS; and within this range, the nomograms for predicting OS and CSS displayed a significantly better performance than SEER stage (Figure 5).

## 4 DISCUSSION

Angiosarcoma is an easily infiltrative tumor with a high rate of local recurrence and metastasis.^13^, ^14^, ^15^ In terms of clinical manifestations and behavior, it demonstrates remarkable heterogeneity and can develop in various anatomical structures. AS also presents a dismal prognosis.^16^ However, due to its rarity and delayed diagnosis, the existing data about clinical characteristics and prognostic factors are limited. Currently, the main criteria for assessing the prognosis of AS are based on the American Joint Committee on Cancer TNM STS staging system.^17^ But TNM staging system still has its limitations. Patients with the same TNM stage but different survival outcomes will be forced to enter the same disease stage, which introduces heterogeneity. Nomograms have been shown to be more accurate in predicting prognosis for many cancer types such as hepatocellular carcinoma and gastric cancer than the TNM staging system.^18^, ^19^, ^20^ Therefore, the goal of this study was to establish and validate nomograms to predict the OS and CSS of AS patients. Data were collected from the SEER database and then analyzed. Next, we included the independent prognostic factors of AS in this study and constructed nomograms. The goal of the nomograms was to predict the 3‐ and 5‐year OS and CSS of AS. The internal and external validation suggested that the models were reliable.

Current treatments for AS include surgery, chemotherapy, and radiotherapy. In addition, targeted therapy and immune therapy are also considered to be promising anticancer therapeutic strategies.^21^, ^22^, ^23^ The results of different treatments vary widely and are affected by many factors, such as tumor location, tumor size, resectability, and tumor type.^13^, ^24^, ^25^ According to previous studies,^26^, ^27^ radical surgery remains the standard therapy for localized AS among different therapeutic approaches. It is still considered to be the most valid method to improve the 5‐year survival rate of AS. Furthermore, surgery was also found to be remarkably related to the survival benefit of patients with AS. For tumors that are smaller and easier to resect, clinicians often choose surgical treatment only. For larger head and neck tumors that are difficult to remove, radiotherapy combined with surgery is the optimal choice.^28^ A similar result was also found in our study: there was a substantial survival advantage among AS patients of localized and regional stages who had undergone surgery.

According to the SEER database, there are three main limitations of the radiotherapy and chemotherapy data: (1) the completeness of the variables; (2) the bias related to unmeasurable reasons for receiving or not receiving radiotherapy/chemotherapy; and (3) the interpretation of the sequence data variables. Furthermore, the radiation dose, radiation volume, radiation modality, radiation technique, chemotherapy regimen, and drug dosage all have an impact on the patients’ survival. In the SEER database, the above details are not recorded. And these deviations would also bring potential errors to the analyses results. However, despite the univariate and multivariate analyses of radiotherapy and chemotherapy lacked statistical significance, we still included these variables in the nomograms. For the data in the database are still available and have been used in many studies.^8^, ^9^, ^11^, ^18^ Moreover, previous study indicated that radiotherapy is effective for inoperable patients with AS and can reduce the risk of postoperative recurrence.^13^ And some retrospective studies have shown that adjuvant radiotherapy may improve the local control and survival of localized AS.^4^, ^29^, ^30^ For patients who have unresectable or recurrent metastatic AS, the main treatment remains chemotherapy.^31^ Thus, both radiotherapy and chemotherapy play essential roles in the treatment of AS and have important clinical significance. In summary, considering the availability of clinical data, clinical evidence, the limitations of the SEER database, and statistical results in Tables 2 and 3, we incorporated treatments including surgery, chemotherapy, and radiotherapy into the construction of the nomograms.

Epidemiological research shows that AS has a similar distribution between sexes. Therefore, sex may not be the most important factor affecting the prognosis of AS. AS can occur at any age, but it is more common in the elderly.^5^, ^13^, ^32^ It has been described that the median age ranges from 60 to 71 years old.^3^ In univariate and multivariate *Cox* regression analysis, age was a significant risk factor for AS patients. The results indicated that younger patients had better outcomes than the elderly patients. Several factors may have accounted for this. First, older AS patients do not tolerate with treatment well, such as surgery, chemotherapy, and radiotherapy. Then, the elderly themselves might have other underlying diseases. For example, heart disease, stroke, and other chronic or long‐term diseases were common in the elderly. Moreover, the cumulative effect of various pathogenic factors increased with age. It also indirectly led to a poor prognosis for the elderly patients. In fact, morbidity and mortality increase with age in many solid tumors.^33^, ^34^ The clinical factors used in the nomogram to predict OS in patients with AS included age. In our study, stage was also identified as an important prognostic factor and it was used for the calculation of nomograms for OS and CSS. Of the three subgroups, patients in the “Distant” group had the worst prognosis, followed by patients in the “Regional” group. Localized AS patients showed a better prognosis. AS mainly occurs in soft tissues and skin. The head and neck are the most common sites for AS, followed by the breast. They can also be located in organs such as the heart.^35^ We divided AS into a “trunk” group and a “limbs” group according to where it occurred. AS in the head and neck was also classified into the “trunk” group. The result was as expected: most of the AS was located in the trunk. As shown in Table 3, tumor site was an independent factor for CSS.

Although the incidence of AS is relatively low, considering its poor prognosis, reports on the prognosis and the establishment of prognostic models are limited, and the main purpose and significance of our research were to construct prognostic models of AS. As far as we know, it has always been a challenge to accurately predict the prognosis of patients with AS. The main advantage of the nomograms is to calculate the prognosis in a personalized way according to the clinicopathological characteristics of the patient and the sarcoma. Nomograms can also visualize complex statistical models. In addition, nomograms can be used in many aspects related to tumors, such as predicting tumor prognosis or metastasis.^8^, ^36^ At present, the use of nomograms has been very extensive. Nomograms have been used in many other tumors including liposarcoma, hepatocellular carcinoma, and endometrial stromal sarcoma.^8^, ^9^, ^37^, ^38^ Therefore, it is necessary and feasible to establish nomograms to accurately predict the prognosis of AS. In the current research, there were already some models of AS. However, these models usually focus on one specific organ.^31^, ^39^, ^40^ Remarkably, our nomograms were constructed for AS that occurred throughout the body. AS accounts for only 1%–2% of STS, and the number of samples is usually small. However, we included a sufficient number of patients with AS by using a public database. Moreover, using DCA to compare the nomograms with the SEER stage, the results showed that our models were clinically useful.

Despite the unique advantages of the model, there are still several inevitable limitations. First, our samples all came from the SEER database. Notably, this database mainly contains information about foreigners. There may be a degree of error in the results. Therefore, the conclusions we presented still await further validation in Chinese patients. Second, the lack of details of treatment protocols and significant laboratory results made it difficult to perform further analyses. Third, this research requires databases other than the SEER database or independent large‐scale data verification support. Fourth, the patients' TNM stage information is incomplete, thus lacking the DCA of nomograms and TNM stage. Last but not least, given the retrospective nature of this study, it bears the inherent limitations of such analyses.

In conclusion, we constructed new and reliable nomograms with the clinical information obtained from the SEER database. As far as we know, this is the first study to build and validate the prognostic nomograms for both OS and CSS in patients with AS that occurred throughout the body. In addition, the DCA results showed that our nomograms had better prediction effects than the SEER stage. The main advantage of nomograms is to visually and individually predict the prognosis of patients with AS. Nomograms may also be used as an important supplement to the current TNM staging system, which offers effective help for both clinicians and patients. Future research can expand the number of cases and further verify its clinical significance and value in multiple cancer centers.

## ETHICAL APPROVAL STATEMENT

Since the Surveillance, Epidemiology, and End Results database contained patient de‐identification information, the approval process of the institutional review board was not required.

## CONFLICT OF INTEREST

The authors made no disclosures.

## Supporting information

Fig S1

Table S1

## Data Availability

Qualified researchers may request data from the Surveillance, Epidemiology, and End Results database. Complete details are available at https://seer.cancer.gov.
