# Supplementary figures and images for: New nomograms to predict overall and cancer‐specific survival of angiosarcoma
Source: Cancer Med. 2021 Nov 16;11(1):74–85. doi: 10.1002/cam4.4425 (PMC8704180; doi:10.1002/cam4.4425)

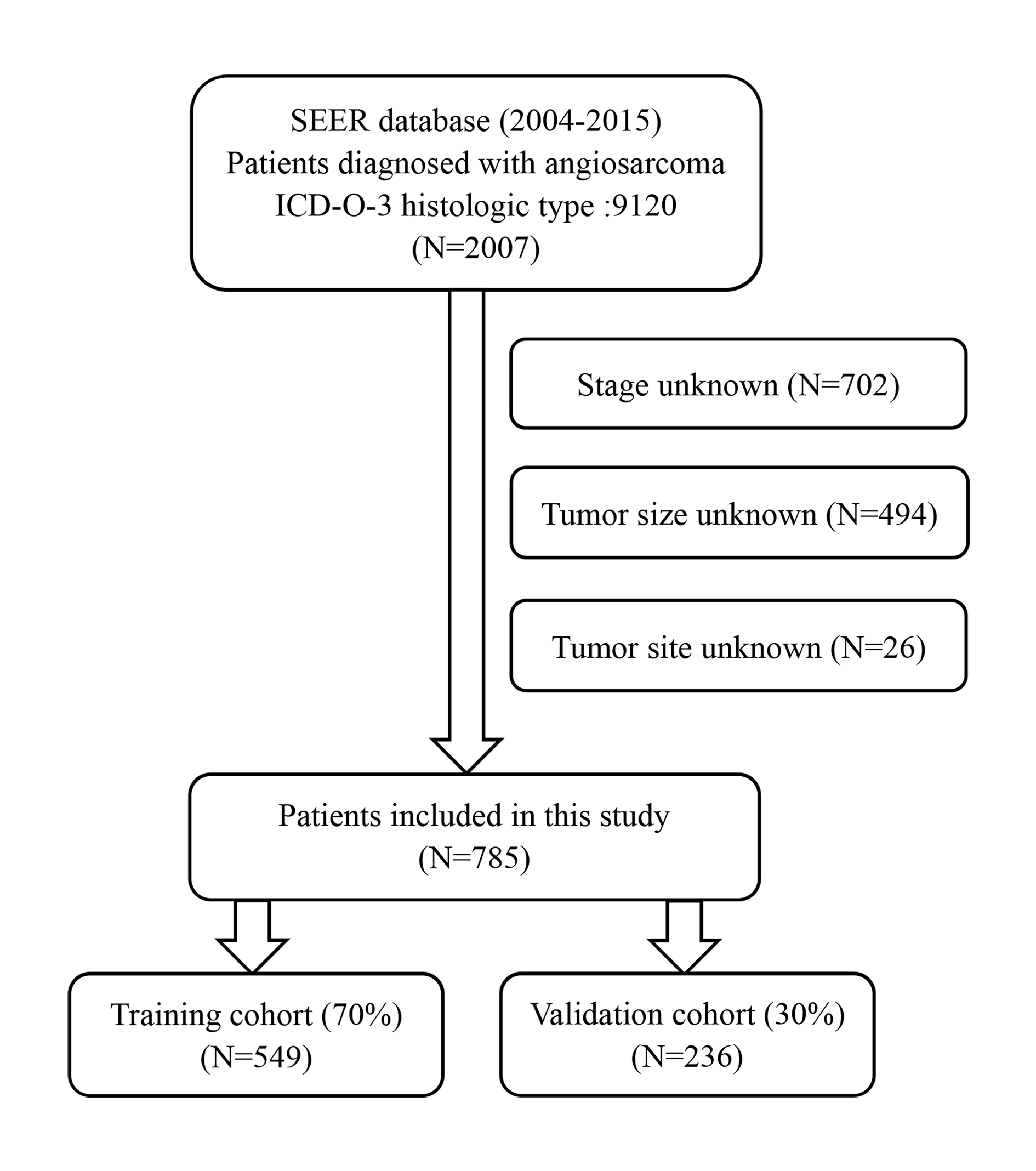

Supplement: Supplementary file 1 — Fig S1 [file CAM4-11-74-s002.tif]
