# Supplementary material for: New nomograms to predict overall and cancer‐specific survival of angiosarcoma
Source: Cancer Med. 2021 Nov 16;11(1):74–85. doi: 10.1002/cam4.4425 (PMC8704180; doi:10.1002/cam4.4425)
Supplement: Supplementary file 2 — Table S1 [file CAM4-11-74-s001.docx]

Table_4_SuppInfo The C-indices for the nomograms in patients with AS.

| Survival | Training cohort | | Validation cohort | |
| --- | --- | --- | --- | --- |
|  | C-index | 95%CI | C-index | 95%CI |
| OS | 0.666 | (0.638,0.694) | 0.712 | (0.668,0.757) |
| CSS | 0.654 | (0.613,0.696) | 0.707 | (0.640,0.774) |

_Abbreviation: AS, angiosarcoma; CSS, cancer-specific survival; OS, overall survival._

**Figure legends**

Figure_6_SuppInfo. Detailed flow diagram of patients screening.
